# Comparison of intraoperative biliary anastomosis stenting technique in living-donor liver transplantation: Review of 41 patients

**DOI:** 10.55730/1300-0144.5394

**Published:** 2022-03-02

**Authors:** Ramazan DÖNMEZ, Şener BALAS, Ufuk Utku GÖKTUĞ, Ertan EMEK, Yaman TOKAT

**Affiliations:** 1 Vocational School of Health Services, Fenerbahçe University İstanbul, Turkey; 2 Department of General Surgery, Dışkapı Yıldırım Beyazıt Training and Research Hospital, University of Health Sciences Ankara, Turkey; 3 Department of General Surgery, Keçiören Training and Research Hospital, University of Health Sciences Ankara, Turkey; 4 Department of General Surgery Medikal Park Göztepe Hospital, İstanbul Turkey; 5 Department of General Surgery Acıbadem Fulya Hospital, İstanbul, Turkey

**Keywords:** Liver, transplantation, biliary, complication

## Abstract

**Background/aim:**

Biliary fistula is one of the most important complications in liver transplantation. Complications can vary from simple local peritonitis to death, and various techniques have been described to prevent them.

In this study, we compared two different stenting methods used in biliary tract anastomosis in living-donor liver transplantation.

**Material and methods:**

We retrospectively analyzed data from 41 living-donor liver transplantations that were performed due to end-stage liver failure between August 2019 and November 2020. Patients were grouped according to the stenting technique used in biliary anastomosis. Postoperative biliary tract complications were investigated.

**Results:**

Biliary fistulas were observed in 2 (7.4%) patients in the internal stent group, while 4 (28.5) fistulas were observed in the external stent group. Biliary tract stricture was observed in 2 (7.4%) patients in the internal stent group, but there was no statistical difference in complications. The preoperative MELD score (p = 0.038*) was found to be statistically significant in regard to developing complications.

**Conclusion:**

Our study did not show the effect of stenting methods used during biliary anastomosis on the development of complications. However, larger randomized controlled studies are needed.

## 1. Introduction

End-stage liver failure is a disease that requires a difficult treatment procedure. Liver transplantation is the most important component of this challenging process. Both cadaveric and living-donor liver transplantations require significant organization and meticulous methods.

The most common and important complications associated with liver transplantation are related to the biliary system. Bile leakage and biliary stenosis are some of the most important problems to be solved. In order to solve these problems, different biliary anastomosis techniques have been studied. In this study, we aimed to compare the results of stenting methods used in bile duct anastomosis applied to 41 living-donor liver transplant patients in our clinic.

## 2. Material and methods

After obtaining approval from the local ethics committee (Yeditepe University Clinical Research Ethics Committee No: 18/2/2021-1390), we retrospectively evaluated data from 44 liver transplantations that were performed at an organ transplant clinic between August 2019 and November 2020. Three deceased-donor liver transplantations were excluded from the study. The patients were grouped according to the type of stent placed in the biliary tract anastomosis. The data examined comprised the demographic data of the patients, the etiology of liver failure, the preoperative Model for End-Stage Liver Disease (MELD) score, the amount of blood used in surgery, the donor’s arterial typing, the number of bile ducts involved in the anastomosis, the number of anastomoses, and the postoperative complications of the recipient.

### 2.1. Stent placement and biliary tract anastomosis technique

**Figure 1:**
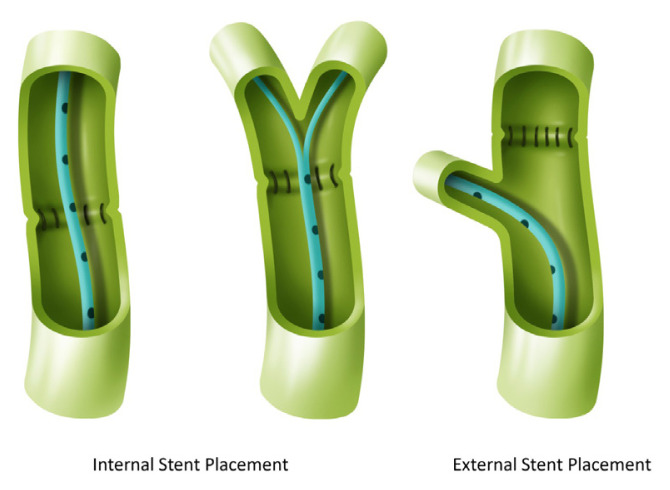
Stent placement.

In all cases, biliary drainage was provided in the grafts by applying the duct-to-channel anastomosis method. Suspension sutures were placed at the 3 o’clock position with 6/0 polydioxanone (PDS) in patients with internal stent placement. The common posterior wall was constructed with interrupted 6/0 PDS sutures, and all nodes were tied outside the anastomosis. After the posterior wall was completed, the tip of a 5F silicone feeding tube was cut as much as the number of channels. Each cut end of a stent was placed in bile ducts separately as part of the anastomosis. The main trunk of the stent was also placed in the recipient’s choledoch. The anterior wall of the anastomosis was completed by placing 6/0 PDS interrupted sutures (Figure 1).

**Figure 2:**
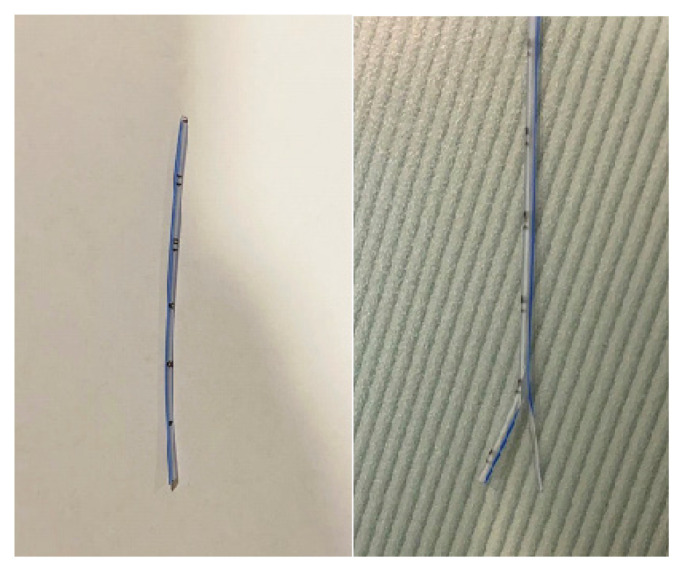
Stents.

In the external stent group, posterior wall reconstruction was achieved by continuous suturing with 6/0 PDS. A 5F silicone feeding tube was inserted through the cystic duct and extended to the distal portion of the common bile duct to provide decompression. The stent was stabilized by surrounding the cystic duct with purse string sutures. The anterior wall was completely closed by placing intermittent sutures with 6/0 PDS. The safety of the anastomosis was checked using cholangiography and methylene blue (Figure 2). All anastomoses were performed under a 3.5× magnification loop.

### 2.2. Statistical analysis

Continuous variables were expressed as the mean ± standard deviation and/or median (min-max), and categorical data were expressed as numbers and percentages. Normality analyses of continuous variables were performed using the Kolmogorov-Smirnov goodness-of-fit test. Variables that fit a normal distribution were compared using the independent-samples t-test, and those that did not were compared using the Mann-Whitney U test. A chi-squared test was used to compare categorical data. Analyses were performed using IBM SPSS version 22.0 (IBM Corporation, Armonk, NY, USA), and the statistical significance level was considered as p < 0.05.

## 3. Results

**Figure 3:**
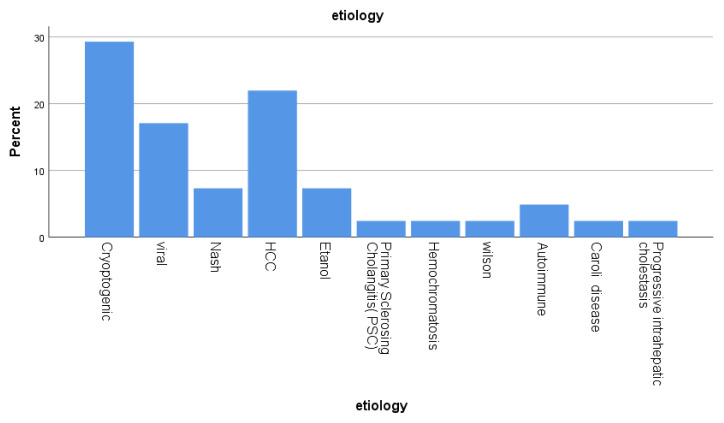
Transplantation indications of patients.

There were 25 (61%) male patients and 16 (39%) female patients. The mean age was 50.1 (7–74) years, and the patients comprised 39 adults and 2 children. Indications for transplantation are listed in Figure 3, and the most common was cryptogenic cirrhosis (12 patients 29.3%). Other common causes were hepatocellular carcinoma and chronic liver disease due to viral hepatitis. The final MELD (MELD-Na) score of the patients calculated before transplantation was 17.47 (8–33).

In preoperative radiological evaluations, donors were assessed according to the Michels classification. Type 1 artery was seen in 29 patients, type 2 was seen in 6 patients, type 3 was seen in 3 patients, type 4 was seen in 2 patients, and type 6 was seen in 1 patient [1,2]. There was one bile duct in 15 transplanted grafts, 2 bile ducts in 22 grafts, 3 bile ducts in 2 grafts, and 4 bile ducts in the other 2 grafts. The biliary anastomosis was performed using the duct-to-duct technique in 37 patients. In four patients, anastomosis was performed using the sheet to duct technique. In 29 patients, a single anastomosis was performed, while two anastomoses were performed in 12 patients.

An internal stent method was used in 27 patients, and an external stent method was used in 14 patients. An average of 1.78 (0–10) units of erythrocytes were transfused in the recipient surgery. External bile drainage catheters were kept open for 1 month and then closed and removed at 3 months. Table 1 shows the demographic and follow-up data of the groups in which both methods were applied. The patients were followed up for a mean of 17.8 (11.8–26.6) months.

Complications that developed for each stenting method are presented in Table 2. No complications were observed in 70.4% (n = 19) of the patients who received an internal stent. Early bile leakage and bilioma were observed in 2 patients (7.4%), who were treated with ERCP and percutaneous drain placement. Biliary stenosis developed in 2 patients during the follow-up period, who were treated with percutaneous transhepatic cholangiography.

Early bile leakage and bilioma developed in 4 (28.5%) patients who underwent external biliary drainage. Biliary anastomosis was renewed by relaparotomy in one of these patients. Two of the remaining three patients were treated with ERCP and percutaneous drainage, while one patient was followed up with percutaneous drainage only. No biliary tract stenosis occurred in this group. There were no deaths related to biliary anastomosis in either group. When the patients were evaluated in terms of complications according to the stent method applied, no significant difference was found between them (p = 0.426*; chi-squared; Table 2).

No significant difference was found in terms of anastomosis and biliary tract numbers in patients with and without complications. However, the final MELD values calculated before transplantation were found to be significantly higher in patients with complications (mean score: 19 (12–33)) and those without complications (mean score: 16 (8–24)) (p = 0.038; Table 3). When controlling for intraoperative transfusion, no significant difference was found in the development of complications (p = 0.229).

## 4. Discussion

The development of fistula or stenosis after bile duct anastomoses is a common complication in living-donor liver transplantation and can sometimes lead to death. Biliary tract complications after liver transplantation significantly affect morbidity and mortality [3]. Ensuring the safety of a bile duct anastomosis is essential to prevent biliary complications such as biliary anastomotic stenosis and bile leakage [4].

In a randomized study, Santosh Kumar et al. examined the effects of placing intraductal stents on biliary duct complications. They defined the involvement of multiple bile ducts and stent placement as independent risk factors in their study [5]. Similarly, Kim et al. suggested that the presence of multiple bile ducts in the graft is a risk factor for biliary complications in living-donor liver transplants. In the reconstruction of more than one bile duct, it appears that mucosal eversion is associated with lower complication rates compared to ductoplasty [6]. In our study, although more than one biliary anastomosis was associated with increased biliary tract complications, no statistically significant correlation could be found.

In a cohort study, Baker et al. analyzed the anatomical variations of the biliary tract and the surgical techniques used in liver transplant patients from nine centers. A lower rate of vascular complications was reported in cases where duct-to-duct biliary reconstruction was performed [7]. In our study, the duct-to-duct method was performed on all patients, and hepatic artery thrombosis was observed in only one patient. Portal vein thrombosis was not observed.

Ishiko et al. retrospectively evaluated biliary tract reconstructions. In bile duct anastomoses with external biliary drainage established by the continuous method, they observed lower rates of leakage and stenosis compared to external stent placement into the cystic duct by the interrupted suturing method. They found that the anastomosis performed by the external stent placement and the continuous suturing technique were more successful [8]. In our patient group, although the rate of bile leakage was higher in patients who underwent external stenting, the development of stenosis was similar to the findings of Ishiko et al.

Icoz et al. analyzed the complications, treatment methods, and outcomes concerning biliary anastomoses in 50 consecutive right-lobe living-donor liver transplant patients. They reported that overall, biliary complications developed at a rate of 30%, and stenosis was observed more commonly in patients with multiple bile ducts [9]. Although our study had fewer patients, our overall complication rate was 31.7%, while our rate of biliary tract-related complications was 19.5%.

Reyes et al. aimed to identify accompanying factors associated with biliary complications. They reported that the diameter of the biliary tract of the recipient and the graft was not related to biliary complications. However, they reported that the difference between the graft diameter and the diameter of the recipient bile duct was important. Furthermore, they reported that arterial ischemia times, arterial complications, and intraoperative blood transfusions might be effective factors [10]. In our study, a relationship was not found between blood transfusions and biliary complications.

Arikan et al. examined the relationship between the number of bile duct anastomoses and biliary complications. They reported that the numbers of bile ducts and anastomoses were unrelated to the complication rates. However, the Child-Pugh score (CPS), graft-to-recipient weight ratio (GRWR), and young donor age were found to be predisposing factors for postoperative biliary complications [11]. According to our data, the MELD-Na scores were associated with early biliary complications.

Hong et al. considered the presence of the following conditions risky for developing biliary complications: small bile duct diameters (≤3 mm), long warm-ischemia time, low GRWR, and not using an external biliary stent [12]. However, the higher rate of fistula occurrence in the external stent group in our study does not support that conclusion. In both the external and internal stenting methods, fistula development was found to be associated with MELD-Na scores. The anastomosis techniques used in the operations were similar in both groups.

Ando et al. reported that in pediatric living-donor liver transplants, biliary reconstruction by the wide-interval interrupted suturing method prevented anastomotic stenoses and bile leakages [13]. In our case series, two pediatric transplants were performed, and biliary anastomoses were performed by interrupted suturing. No complications occurred, but we do not consider the result significant because the number is too small.

Verdonk et al. reported that bile leakages occur in approximately 5%–7% of organ transplant cases and that such leakages occur at the site of the anastomosis or around the T-tube stent [14]. T-tube stenting was not preferred in our study. Gruttadauria et al. performed an anastomosis on the cystic and common bile ducts by placing a 6-cm 5Fr silicone catheter extending through the trans-papillary route in a graft with two bile ducts. They reported that living donors with multiple bile ducts should not be rejected [15].

Jung et al. examined effective factors in reducing biliary complications in tension-free anastomosis in living-donor liver transplants. They reported that the effective factors were an adequate blood supply to the bile duct, careful use of the anastomosis technique, performing mucosal eversion, ensuring less fibrosis to ensure better approximation, and the use of internal or external stents [16]. We did not prefer mucosal eversion in our technique. However, we meticulously performed the remaining components mentioned.

## 5. Conclusions

Bile duct complications have a significant impact on morbidity and mortality in living-donor liver transplantation. Therefore, establishing the safety of a bile duct anastomosis is essential to prevent biliary complications. Factors that reduce the risk of complications are adequate blood flow to the anastomosis, the anastomotic technique used, the correct use of stents, and internal stenting.

According to our data, a significant correlation of biliary complications was observed with MELD-Na scores. When we compare biliary drainage techniques, we cannot say that either stenting method is proportionally more advantageous in terms of early complications. A possible advantage can be attributed to the passage of the catheter through the anastomosis and early drainage of bile. We believe that the most reliable way to compare the two biliary drainage methods will be to conduct large-scale, randomized, and prospective studies.

## Figures and Tables

**Table 1:** Comparison of patient groups with internal and external stent in terms of demographic and some clinical characteristics.

Complications		Internal stent (n = 27)	External stent (n= 14)	
**Age (year ) (mean**±S**tdv)**		51.81 ± 11.78	54.64 ± 13.32	0.490*
**BMI (kg/m^2^) (mean**±S**tdv)**		27.54 ± 4.09	26.16 ± 4.04	0.309*
**Length of hospital stay (days)**		13.30 ± 7.02	17.79 ± 8.19	0.074*
**Gender (n, %)**	**Female**	8 (28.6%)	7 (50.0%)	0.199**
	**Male**	19 (70.4%)	7 (50.0%)	
**Exitus (n, %)**	**(+)**	26 (96.3%)	13 (92.9%)	0.628**
	**(−)**	1 (3.7%)	1 (7.1%)	
**Number of erythrocyte transfusion unit**	**0**	15 (55.6%)	3 (21.4%)	0.188*
	**1**	3 (11.1%)	2 (14.3%)	
	**2**	3 (11.1%)	4 (28.6%)	
	**≥3**	6 (22.2%)	5 (35.7%)	
**Number of bile ducts**	**1**	10 (37.0%)	5 (35.7%)	0.726**
	**2**	14 (51.9%)	8 (57.1%)	
	**3**	1 (3.7%)	1 (7.1%)	
	**4**	2 (7.4%)	0 (0.0%)	
**Number of anastomosis**	**1**	18 (69.2%)	11 (78.6%)	0.528**
	**2**	8 (30.8%)	3 (21.4%)	
**Total**		27 (100.0%)	14 (100.0%)	

* Independent samples t-test

** Chi-square tes

**Table 2:** Complications after recipient operation.

Complication	Internal Stant (n = 27)	Ekternal Stant (n = 14)	p
(−)	19 (70.4%)	9 (64.3%)	0.426*
Hepatic artery thrombosis	1 (3.7%)	0 (0.0%)	
Acute rejection	0 (0.0%)	1 (7.1%)	
Early postop bleeding	2 (7.4%)	0 (0.0%)	
Biliary fistula + bilioma	2 (7.4%)	4 (28.5%)	
Late biliary stricture	2 (7.4%)	0 (0.0%)	
Exitus **/***	1 (3.7%)	1 (7.1%)	
Total	27 (100.0%)	14 (100.0%)	

* Chi-square test

** (Postoperative Covid-19 infection)

*** (Postoperative myocardial infarction after discharge from hospital)

**Table 3:** Comparison of anastomosis and biliary tract numbers in patients with and without complications.

	Complication (−) (n = 28)	Complication (+) (n = 13)	p
**Number of biliary anastomosis [median (min-max)]**	1 (1–2)	1 (1–2)	0.424*
**Number of bile ducts [median (min-max)]**	2 (1–4)	2 (1–4)	0.249*
**Preop Meld-Na Score [median (min-max)]**	16 (8–24)	19 (12–33)	**0.038** *

* Mann-Whitney U Test
